# Protective Cancer Vaccine Using Genetically Modified Hematopoietic Stem Cells

**DOI:** 10.3390/vaccines6030040

**Published:** 2018-07-06

**Authors:** Xiaofang Xiong, Jugal Kishor Das, Jianyong Song, Bing Ni, Xingcong Ren, Jin-Ming Yang, Jianxun Song

**Affiliations:** 1 Department of Microbial Pathogenesis and Immunology, Texas A&M University Health Science Center, College Station, TX 77843, USA; xxiong@tamhsc.edu (X.X.); das@tamhsc.edu (J.K.D.); 2 Institutes of Irradiation/Immunology, The Third Military Medical University, Chongqing 400038, China; songjy6415@163.com (J.S.); nibingxi@126.com (B.N.); 3 Department of Pharmacology, The Pennsylvania State University College of Medicine, Hershey, PA 17033, USA; xxr1@psu.edu (X.R.); juy16@psu.edu (J.-M.Y.)

**Keywords:** hematopoietic stem cells, T cells, immunotherapy, melanoma, mouse model

## Abstract

Hematopoietic stem cells (HSCs) yield both the myeloid and lymphoid lineages of blood cells and can be reprogrammed into tumor antigen (Ag)-specific CD8^+^ cytotoxic T lymphocytes (CTLs) to prevent tumor growth. However, the optimal approach for differentiating tumor Ag-specific CTLs from HSCs, such as HSC-CTLs, remains elusive. In the current study, we showed that a combination of genetic modification of HSCs and in vivo T cell development facilitates the generation of Ag-specific CTLs that suppressed tumor growth. Murine HSCs, which were genetically modified with chicken ovalbumin (OVA)-specific T cell receptor, were adoptively transferred into recipient mice. In the following week, mice were administered with intraperitoneal injections of an agonist α-Notch 2 antibody and cytokines (rFlt3L and rIL-7) three times. After another two weeks, mice received a subcutaneous inoculation of B16-OVA melanoma cells that express OVA as a surrogate tumor Ag, before the anti-tumor activity of HSC-derived T cells was assessed. OVA-specific CTLs developed in vivo and greatly responded to OVA Ag stimulation ex vivo. In addition, mice receiving genetically modified HSCs and in vivo priming established anti-tumor immunity, resulting in the suppression of tumor growth. These results reported in this present study provide an alternative strategy to develop protective cancer vaccines by using genetically modified HSCs.

## 1. Introduction

In the last few years, the growing advancements in cellular immunology and tumor–host immune interactions has led to promising development of various immunotherapeutic approaches, including the blockade of immune checkpoints, induction of cytotoxic T lymphocytes (CTLs), adoptive cell transfer (ACT)-based therapy and modulation of tumor microenvironment (TME) to facilitate CTL activity. Many studies have demonstrated encouraging results. For example, ACT of tumor-infiltrating T lymphocytes (TILs) that were activated and expanded ex vivo has shown positive clinical outcomes in patients with metastatic malignancy [1]. In patients with refractory tumors, TIL therapy has response rates of approximately 50% and substantial survival benefits even with patients that have failed other immunotherapies, including checkpoint inhibitors or cytokine-based therapy [2]. Nevertheless, in spite of the impressive progress in the field of T cell-based therapy, the clinical outcome remains limited or less satisfactory due to a variety of factors that lessen anti-tumor immunity, which include limited in vivo T cell expansion and persistence, tumor escape mechanisms, target antigen (Ag) selection, TME, T cell trafficking to tumor sites, etc. Successful strategies to solve these problems should drastically progress cancer immunotherapy and thus, are urgently desired.

Tumor Ag-specific CD8^+^ T cells bearing with long telomeres are optimal in ACT-based therapies because these CD8^+^ T cells have better proliferation, survive for longer than short-telomere cells and have a greater response to homeostatic cytokines. However, the obstacles preventing us from obtaining large numbers of such cells from patients restricts the use of ACT-based therapies. Up to now, stem cells are the sole resource that are accessible to produce such optimal CD8^+^ T cells. Induced pluripotent stem cells (iPSCs) provides an opportunity to generate patient- or disease-specific stem cells [3,4,5]. Our previous studies indicated that in vivo programming of Ag-specific iPSC-T cells can be used for cell-based therapies of cancers and autoimmune disorders [6,7,8,9]. However, more studies are needed to verify the safety of iPSCs, including confirmation of whether they have less or no immunogenicity [10,11] because iPSCs are normally generated from somatic cells by the transduction of various transcription factors, including the oncogene of c-Myc.

Hematopoietic stem cells (HSC), which are physiologically derived from bone marrow, peripheral blood or umbilical cord blood, have the ability to self-renew and differentiate into tumor Ag-specific CD8^+^ T cells. Optimizing an approach to generating naive tumor Ag-specific CD8^+^ T cells with long telomeres will greatly help the development of stem cell-derived cancer vaccines. In the current study, we genetically modified murine HSCs with chicken ovalbumin (OVA)-specific T cell receptor (TCR), which were adoptively transferred into recipient mice. In the following week, mice were administered with intraperitoneal (*i.p.*) injections of an agonist α-Notch 2 antibody and cytokines (rFlt3L and rIL-7). After another week, mice received a subcutaneous (*s.c.*) inoculation of B16-OVA melanoma cells that express OVA as the surrogate tumor Ag. We found that OVA-specific HSC-CTLs developed in vivo and greatly responded to OVA Ag stimulation ex vivo. In addition, mice receiving genetically modified HSCs and in vivo priming had OVA-specific HSC-CTLs dramatically infiltrating into their melanoma tissue, which significantly inhibited tumor growth and improved animal survival. Our strategy to generate naive tumor Ag-specific HSC-CTLs may be applied for the development of protective cancer vaccines.

## 2. Results and Discussion

### 2.1. Generation of OVA-Specific TCR Gene-Transduced HSCs

**Figure 1:**
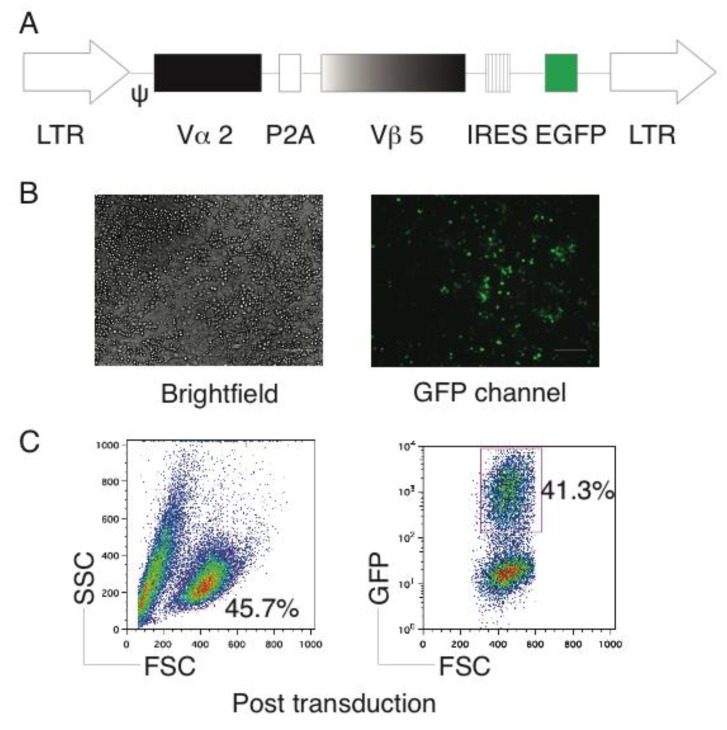
Genetic modification of HSCs with Ag-specific TCR. Bone marrow-derived CD117^+^ Lin^-^ HSCs from C57BL/6 mice were transduced with the retroviral constructs Mig-TCR, which contains ovalbumin (OVA)-specific TCR (Mig-TCR) and enhanced Green Fluorescent Protein (GFP). (**A**) Schematic representation of the retroviral construct Mig-TCRVα2-2A-TCRVβ5 expressing OVA-specific TCR. Ψ, packaging signal; 2A, picornavirus self-cleaving 2A sequence; LTR, Long terminal repeats. (**B**) TCR-transduced HSCs were visualized by fluorescence microscopy (scale bars: 50 μm). (**C**) TCR-transduced HSCs were analyzed by flow cytometry (left) and GFP^+^ cells were sorted by a high-speed cell sorter (right). Data are representative of three independent experiments.

The retroviral vector Mig [12] was used in this study, which contains OVA-specific TCR Vα2 and Vβ5 genes (Figure 1A). After the retroviral transduction, the green fluorescent protein (GFP) expression was visualized by fluorescent microscopy (Figure 1B), before we sorted for GFP^+^ cells (Figure 1C). We retained GFP^+^ HSCs derived from the bone marrow of C57BL/6 mice (Thy 1.2^+^) on the feeder layers of irradiated SNL76/7 cells, which maintained HSC multipotency and self-renewal as previously described [6].

### 2.2. TCR Gene-Transduced HSCs Differentiated into Ag-Specific CD8^+^ T Cells In Vivo

**Figure 2:**
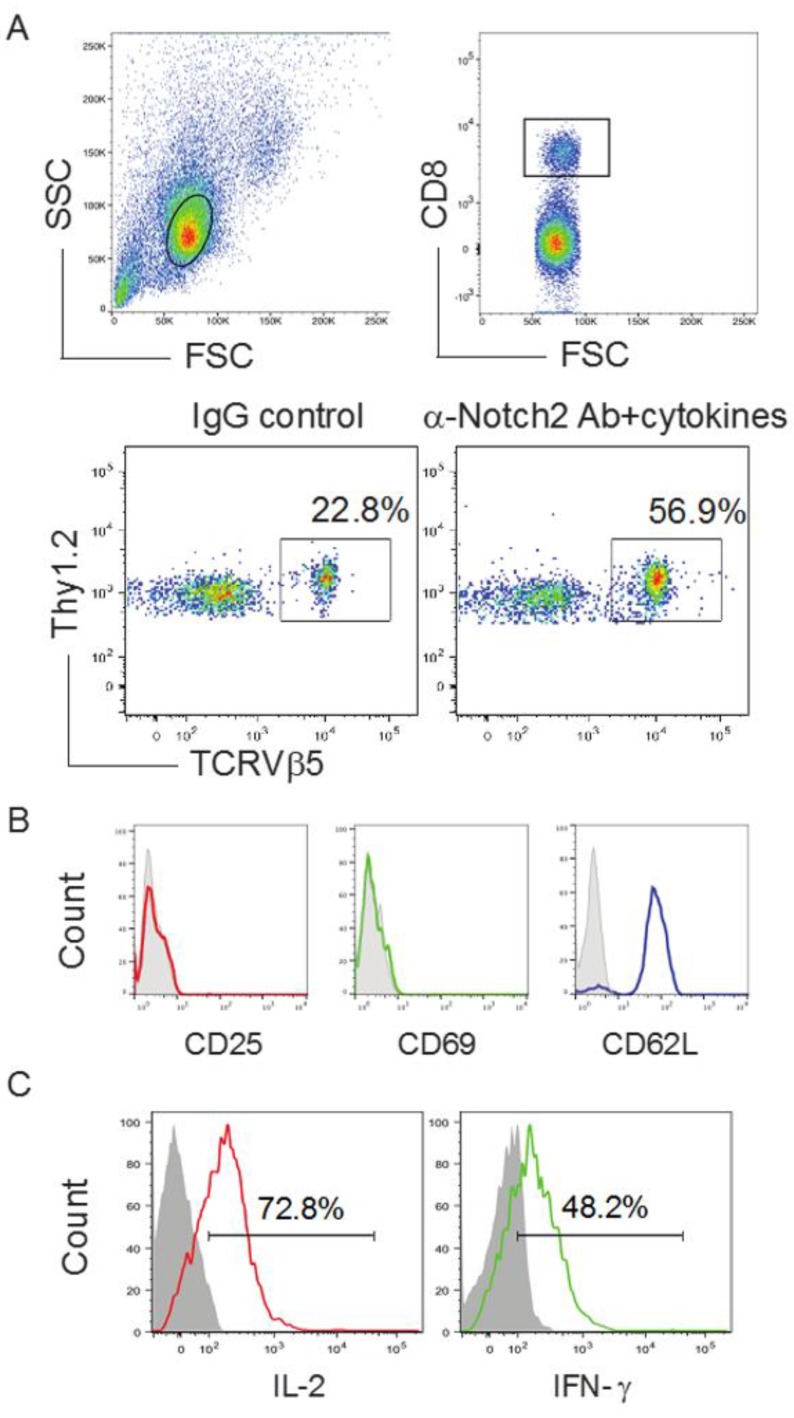
Development of Ag-specific CD8^+^ T cells by genetic modification of HSCs following in vivo priming. TCR gene-transduced HSCs (GFP^+^) in PBS were sorted, before being adoptively transferred into Thy1.1 congenic mice. On the following week, mice were *i.p.* injected with agonistic α-Notch2 Ab, rIL-7 and rFlt3L or a mouse IgG/PBS control. (**A**) After another two weeks, Thy1.2^+^ TCRVβ5^+^ cells from the pooled LNs and spleen were analyzed by flow cytometry, after gating on CD8^+^ T cell population (upper panels). A representative image from mice receiving the HSCs transduced with the Mig-TCR is shown, which was obtained after IgG control or agonistic α-Notch2 Ab, rIL-7 and rFlt3L protein injections. (**B**) Expression of CD25, CD69 and CD62L was analyzed by flow cytometry, after gating on CD8^+^ Thy1.2^+^ TCRVβ5^+^ T cells from the pooled LNs and spleen (dark lines; shaded areas indicate isotype controls). Data are representative of three independent experiments. (**C**) IL-2 and IFN-γ production (dark lines; shaded areas indicate isotype controls). The pooled LNs and spleen were stimulated with OVA_257–264_ peptide and analyzed by intracellular cytokine staining, after gating on Thy1.2^+^ TCRVβ5^+^ cells. Data are representative of three independent experiments.

Our previous report showed that TCR gene-transduced iPSCs developed into Ag-specific CD8^+^ T cells in vivo [6]. We examined whether the OVA TCR gene-transduced HSCs have the ability to differentiate into OVA-specific CD8^+^ T cells in vivo. We adoptively transferred the OVA TCR gene-transduced GFP^+^ HSCs into the recipient congenic mice (Thy 1.1^+^). We *i.p.* injected agonistic α-Notch2 Ab [13,14] and recombinant cytokines (e.g., rIL-7 and rFlt3L) three times a week to boost the development of OVA-specific HSC-CD8^+^ T cells. After another two weeks, we examined Thy1.2^+^ TCRVβ5^+^ cells in the lymph nodes (LNs) and spleen, gating on CD8^+^ T cell population. The approach resulted in capturing approximately 57% of OVA-specific HSC-CD8^+^ T cells (Figure 2A), which did not express CD25 and CD69, but expressed CD62L (Figure 2B). This indicated that the management with in vivo Notch signaling stimulates the development of naive Ag-specific HSC-CD8^+^ T cells. Thy1.2^+^ TCRVβ5^+^ cells from the pooled LNs and spleen responded to OVA Ag stimulation and produced large amounts of IL-2 and IFN-γ (Figure 2C). These data obviously show the in vivo development of functional Ag-specific HSC-CD8^+^ T cells using in vivo Notch signaling.

### 2.3. In Vivo Priming of Ag-Specific HSC-CD8^+^ T Cells

**Figure 3:**
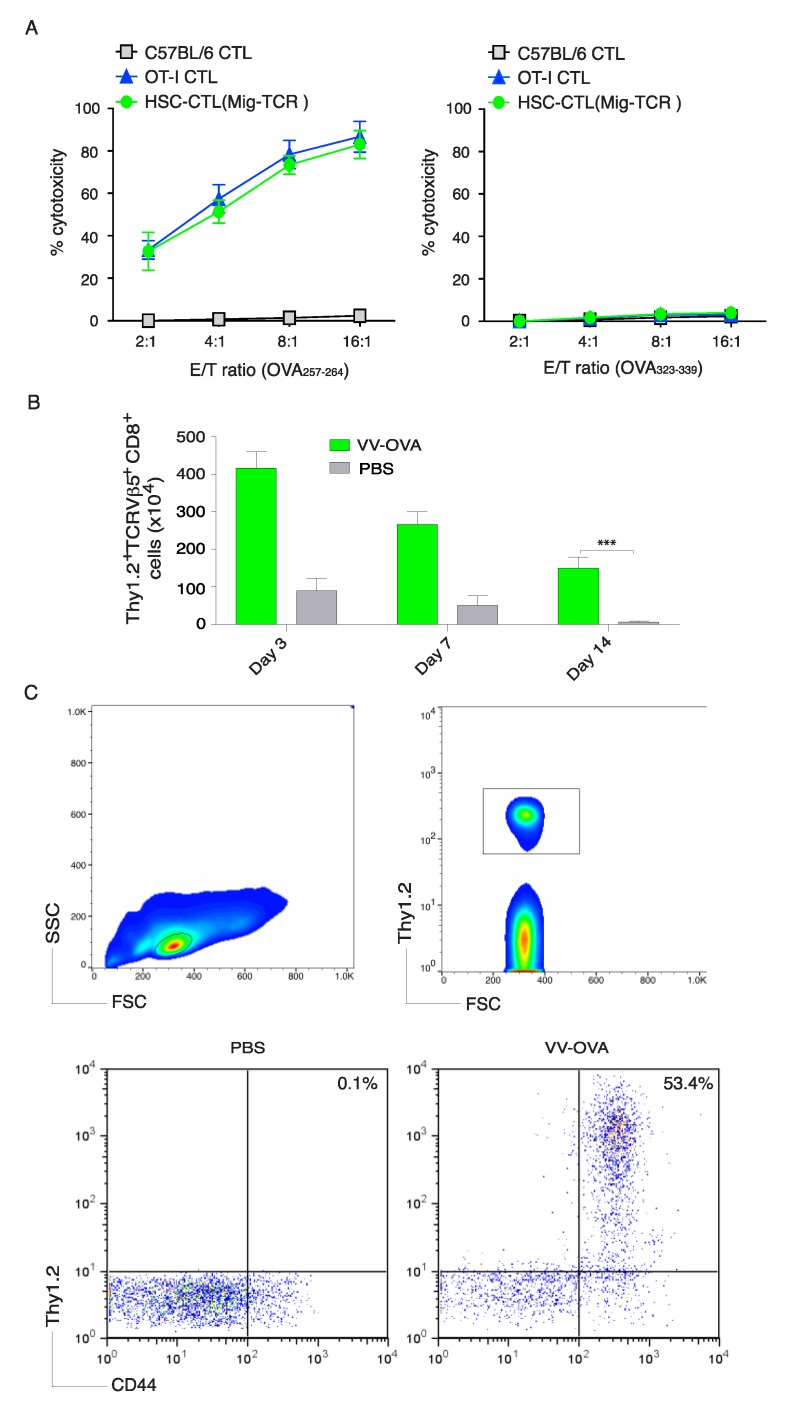
Ag-specific HSC-CTLs persist in vivo. After the development of Ag-specific CD8^+^ T cells by genetic modification of HSCs a in vivo priming as described in Figure 2, CD8^+^ Thy1.2^+^ TCRVβ5^+^ T cells from the pooled LNs and spleen were sorted and an in vitro cytotoxicity assay was performed. In some experiments, mice were infected *i.p.* with VV-OVA or PBS. After various days, OVA-specific T cells from the pooled LNs and spleen were analyzed. Five mice were used for each time point. (**A**) In vitro cytotoxicity assay. The HSC-CTLs, OVA_257–264_-specific CTLs from OT-I TCR Tg mice or non-specific CTLs from C57BL/6 mice were added at different effector to target cell (E:T) ratios. Analysis was performed after a 12 h incubation period. Data are shown as mean ± sd from three wells and are representative of three experiments. (**B**) On days 3, 14 and 21, Thy1.2^+^ TCRVβ5^+^CD8^+^ T cells were counted from the pooled LNs and spleen. Data are the mean number of Thy1.2^+^ TCRVβ5^+^CD8^+^ cells ± sd from five individual mice and are representative of three experiments (* *p* < 0.05, Student’s unpaired *t*-test). (**C**) At day 32, the percentage of Thy1.2^+^ CD44^+^ T cells was analyzed by flow cytometry, after gating on live CD8^+^ T cells in the spleen (upper panels). Results are representative of three experiments.

To further determine the functionality of Ag-specific HSC-CD8^+^ T cells created by the in vivo approach, we performed an in vitro cytotoxicity assay. Ag-specific HSC-CD8^+^ T cells and CD8^+^ T cells from OT-I TCR transgenic mice exhibited comparable OVA_257–264_ Ag-specific cytotoxicity and were not be able to respond to non-specific OVA_323–339_ stimulation (Figure 3A). These data suggest that OVA-specific HSC-CD8^+^ T cells created by the in vivo approach have cytotoxic functions.

To define if OVA-specific HSC-CD8^+^ T cells created in vivo could generate CTL persistence, we *i.p.* infected mice that had received OVA TCR gene-transduced HSCs and in vivo development as described in Figure 2 with recombinant vaccinia viruses expressing the gene for OVA (VV-OVA). On various days (3, 7 and 14), the OVA-specific T cell response was determined from the pooled LNs and spleen. The OVA Ag-specific HSC-CD8^+^ T cells created by the in vivo approach proliferated and expanded (Figure 3B), which also indicated that Ag-specific HSC-CD8^+^ T cells are functional. Of note, on day 35 post-infection of VV-OVA, OVA Ag-specific HSC-CD8^+^ T cells developed memory CTLs (CD44^+^ TCRVβ5^+^) from the pooled LNs and spleen of mice, which was found after gating on CD8^+^ cells (Figure 3C). Collectively, these results strongly support the conclusion that the Ag-specific HSC-CD8^+^ T cells have the ability to induce a persistent Ag-specific immune response.

### 2.4. Genetically Modified HSCs Following In Vivo Priming Induced CTL Infiltration in the Tumor Tissue

**Figure 4:**
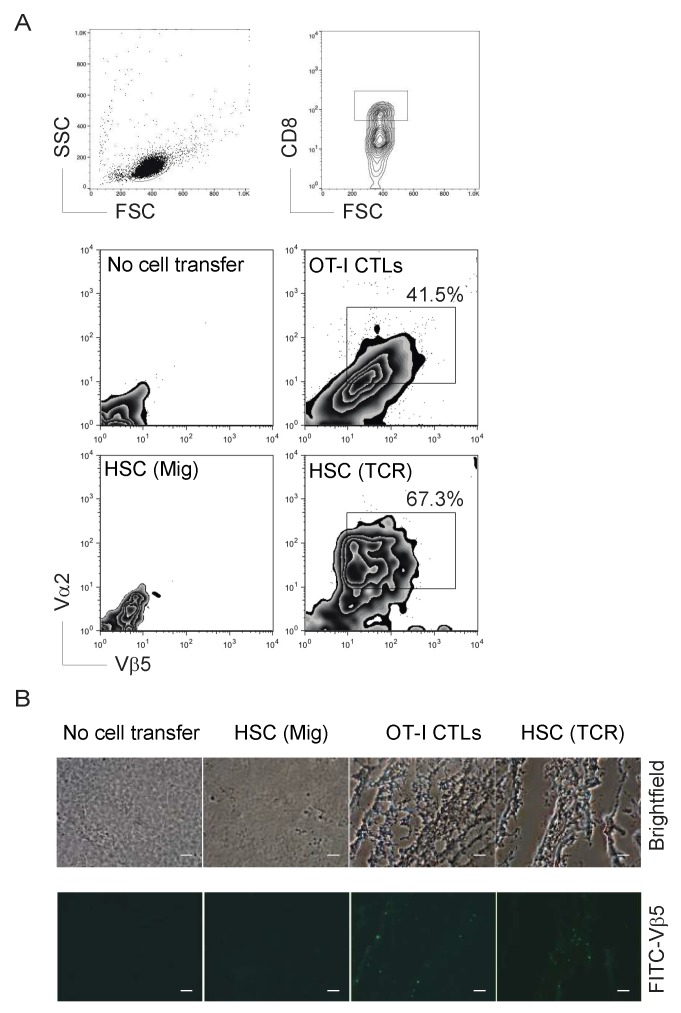
Immunization using genetic modification of HSCs following in vivo priming results in tumor-reactive T cell infiltration. After the development of Ag-specific CD8^+^ T cells by genetic modification of HSCs following in vivo priming as described in Figure 2, the flank regions of mice were *s.c.* injected withB16-OVA melanoma cells that express OVA Ag recognizable by the OVA_257–264_-specific CTLs. Mice were subsequently *i.v.* infected with VV-OVA and were *i.p.* administered rIL-2 after tumor inoculation twice per day for 3 days. On days 14 and 20 after tumor challenge, tumor tissues were examined for tumor-reactive T cell infiltration. (**A**) Single-cell suspensions from tumor tissues were analyzed for expression of TCRVα2^+^ and TCRVβ5^+^ by flow cytometry, after gating on the CD8^+^ population (upper panels). Data are representative of three independent experiments (*n* = 6). (**B**) Immunohistological staining (scale bars: 20 μm). OVA-specific TCRVα2^+^ CD8^+^ T cells in green infiltrated into tumor tissues in black. Data shown are representative of three identical experiments.

To show that the Ag-specific HSC-CD8^+^ T cells created by the in vivo approach have the ability to induce tumor Ag-specific CTL persistence in a physiologically and clinically relevant setting, we used a murine model of melanomas. We performed *i.v.* adoptive transfer of OVA TCR gene-transduced HSCs into the recipient congenic mice, before we *i.p.* injected agonistic α-Notch2 Ab and recombinant cytokines three times a week as described in Figure 2. Two weeks later, we *s.c.* injected B16-OVA melanoma cells into the flank region. To generate better therapeutic effects, we also *i.v.* infected mice with VV-OVA after tumor inoculation and administered rIL-2 *i.p.* twice per day for 3 days [15]. Three weeks after the tumor challenge, we observed a large number of tumor Ag-reactive CD8^+^ T cells infiltrating into melanoma tissues in mice receiving TCR gene-transduced HSCs and in vivo development, which was similar to the mice receiving CD8^+^ T cells from OT-I TCR transgenic mice by flow cytometry (Figure 4A) and immunohistological staining (Figure 4B). These findings indicate that the ACT of TCR gene-transduced HSCs and in vivo development can develop tumor Ag-specific CTL infiltration in tumor tissue.

### 2.5. Immunization Using Genetically Modified HSCs Following In Vivo Priming Protected Mice from Tumor Growth

**Figure 5:**
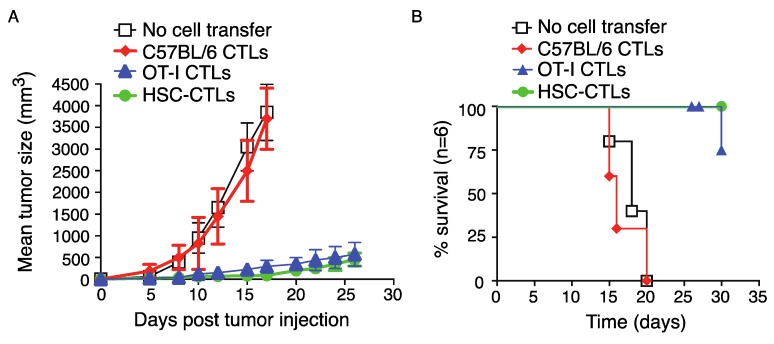
Immunization using genetic modification of HSCs following in vivo priming suppress tumor growth. T cell development and tumor injection were performed as described in Figure 4. (**A**) Tumor growth was monitored over time. Data are shown as mean tumor size ± sd from six individual mice and are representative of three experiments. (**B**) Mouse survival was assessed over 30 days (Kaplan–Meier survival analysis). Data are representative of three independent experiments (*n* = 6).

Large numbers of tumor Ag-specific CTLs infiltrating into the tumor tissue can result in the suppression of tumor growth. Mice that received TCR gene-transduced HSCs and in vivo development were well protected in terms of length of survival. In general, mice in control groups start to succumb from two weeks after melanoma challenge. However, all mice that received TCR gene-transduced HSCs and in vivo development suppressed tumor growth (Figure 5A) and survived the 35-day period of observation (Figure 5B), which was similar to the mice receiving CD8^+^ T cells from OT-I TCR transgenic mice.

### 2.6. Immunization Using Genetically Modified HSCs Following In Vivo Priming Induced the Generation of Tumor Ag-Specific T Cell Memory

**Figure 6:**
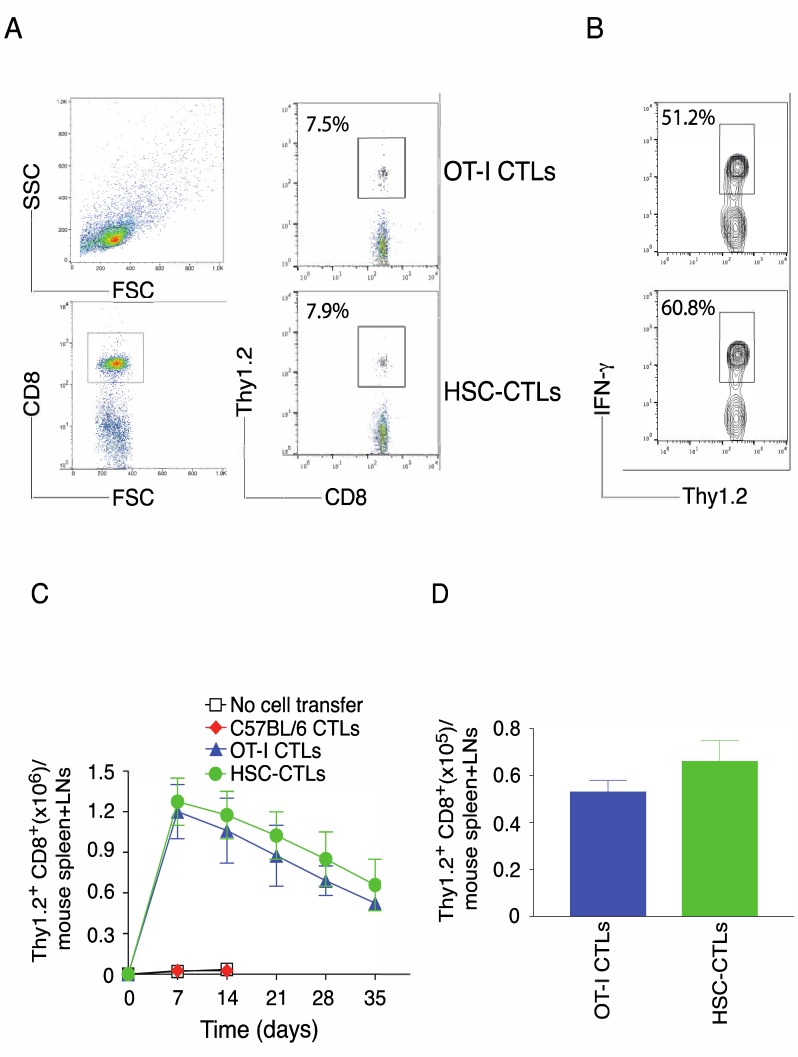
Immunization using genetic modification of HSCs following in vivo priming induces strong Ag-specific T cell memory. T cell development and tumor injection were performed as described in Figure 4. After various days, memory progenitors or memory T cells from the spleen and LNs were analyzed. Five mice were used for each time point. (**A**) The frequencies of Thy1.2^+^ at day 35, gating on CD8^+^ cells (left panels). Data are representative of three independent experiments (*n* = 5). (**B**) Functional analysis using IFN-γ at day 35. Splenocytes were stimulated with OVA peptide for intracellular IFN-γ staining, gating on Thy1.2^+^ cells. Data are representative of three independent experiments (*n* = 5). (**C**) Numbers of CD8^+^ Thy1.2^+^ T cells on indicated day. Data are represented as the mean ± SEM. (**D**) The absolute numbers of CD8^+^ Thy1.2^+^ T cells at day 35. Data are represented as the mean ± SEM from three independent experiments (*n* = 5) (*p* > 0.05, Student’s unpaired *t*-test).

The generation of tumor Ag-specific T cell memory is critical for the development of cancer vaccines. We examined the generation of tumor Ag-specific CTL memory after immunization using genetically modified HSCs following in vivo priming and melanoma challenge. On day 35 post-tumor challenge, CTL memory was determined from the pooled LNs and spleen of mice after gating on CD8^+^ cells. OVA Ag-specific HSC-CD8^+^ cells and CD8^+^ T cells from OT-I TCR transgenic mice developed similar numbers of memory T cells (7.9% vs. 7.5%), which was determined by analyzing CD8^+^ Thy1.2^+^ cells (Figure 6A) and intracellular IFN-γ production after gating on Thy1.2^+^ populations (Figure 6B). Specifically, OVA Ag-specific HSC-CD8^+^ cells and CD8^+^ T cells from OT-I TCR transgenic mice developed a similar kinematic profile of memory T cell development according to the analysis of CD8^+^ Thy1.2^+^ cells on various days (Figure 6C) and equivalent number of memory CTLs on day 35 (Figure 6D). Collectively, these results strongly support the conclusion that the immunization of TCR gene-transduced HSCs have the ability to generate a persistent anti-tumor immune response.

In conclusion, tumor TCR gene-transduced HSCs could serve as cancer vaccines. After immunization by adoptive transfer, these HSCs are capable of developing highly active tumor Ag-specific CD8^+^ cells that suppress tumor growth and acquire tumor Ag-specific CTL memory.

However, there are limitations preventing us from moving forward with this development. First, the development of effective cancer vaccines depends on the identification of tumor injection Ags, which can be used to clone TCR and TCR transduction. It is hard to identify the targeting tumor Ags in many malignancies. Second, HSCs are pluripotent adult stem cells that can differentiate into myeloid or lymphoid progenitors. HSCs need a suitable microenvironment to maintain their stem cell properties. In bone marrow, the microenvironment is extremely complicated as HSCs are bound by bone matrix and various cells, such as fibroblast, adipocyte, macrophage and endothelial cells, which generate different cytokines and growth factors. In fact, the characterization of the optimal conditions for the in vitro culture of HSCs remains unclear. Third, since TCR transduced HSCs need up to three weeks to develop into fully differentiated T cells, there are possibilities to enhance this development. Cytokine administration (IL-7 or IL-15) and agonist Notch signaling can promote T cell differentiation. An optimal in vivo priming may be useful for the translation of the studies to the treatment of cancer patients.

Of note, the GFP-reported gene incorporated into the TCR-vector theoretically provides a convenient way to track the TCR-engineered HSCs and their progeny cells, with GFP^−^ cells possibly able to be used as a congenial internal control. However, after adoptive transfer, the GFP^+^ HSCs differentiated into T cells and did not maintain GFP expression in our experiments. Furthermore, in mouse models, it usually takes 6–8 weeks for adoptively transferred HSCs to fully reconstitute the T cell compartment. In addition to agonist anti-Notch antibody and rFlt3L, the administration of VV-OVA and rIL-2 was used to optimize T cell development of HSCs in vivo. In the current study, Ag-specific HSC-CTLs were generated in 3 weeks and the response to the tumor challenge was able to be measured in 2 weeks.

## 3. Materials and Methods

### 3.1. Ethics Statement

All animal experiments were approved by the Texas A&M University Animal Care Committee and were conducted in compliance with the guidelines of the Association for the Assessment and Accreditation of Laboratory Animal Care.

### 3.2. Cells and Mice

CD117^+^ HSCs from the bone marrows of C57BL/6 mice were cultured on SNL feeders [6] and transduced with retroviral vectors expressing GFP alone or GFP with OVA_257–264_-specific TCR (GFP-TCR). The OVA-expressing B16 (B16-OVA) and B16 melanoma cell lines were purchased from the ATCC (Manassas, VA, USA). Both male and female OT-I TCR transgenic, C57BL/6 and Thy1.1 congenic mice (B6.PL-*Thy1a*/CyJ) mice (4–6-week-old) were purchased from The Jackson Laboratory (Bar Harbor, ME, USA).

### 3.3. Cell Culture

HSCs were maintained on feeder layers of irradiated SNL76/7 cells as previously described [6].

### 3.4. Antibodies Used

OVA_257–264_ (SIINFEKL) peptide was purchased from the American Peptide Company (Sunnyvale, CA, USA). PE, PE/Cy7, PerCP, PerCP/Cy5.5, or APC conjugated Thy1.2 (Clone 53-2.1), TCRVβ5 (MR9-4), TCRVα2 TCR (B20.1), CD44 (Clone IM7), IFN-γ (Clone XMG1.2) and IL-2 (JES6-5H4) were purchased from Biolegend (San Diego, CA, USA). Mouse α-Notch2 Ab was kindly provided by Dr. Hideo Yagita (Juntendo University School of Medicine, Japan) [13,14].

### 3.5. Retroviral Transduction

Retroviral transduction was performed as described previously [6]. The expression of GFP was determined by flow cytometry gating on GFP^+^ cells. GFP^+^ HSCs were sorted by cell sorting using a high-performance cell sorter (BD FACSAria II, San Jose, CA, USA).

### 3.6. Adoptive Cell Transfer

GFP^+^ HSCs (3 × 10^6^) and controls were washed and re-suspended in cold PBS before *i.v.* injection into mice through the tail vein. In the following week, mice were administered with an *i.p.* injection of 0.25 mg of agonistic agonist α-Notch 2 antibody, 5 μg of mouse rIL-7 and 10 μg of mouse rFlt3L (PeproTech, Rocky Hill, NJ, USA) or a mouse IgG/PBS control three times. In another two weeks, CD8^+^ T cell development in the lymph nodes and spleen was determined by flow cytometry. The development of OVA-specific TCRVβ5^+^ CD8^+^ T cells in the lymph nodes and spleen was determined by flow cytometry.

### 3.7. Ex Vivo Peptide Stimulation Assay

OVA-specific HSC-CTLs recovered from the adoptively transferred mice were evaluated for their Ag reactivity by an ex vivo stimulation assay. Briefly, CD8^+^ cells were negatively selected from pooled LNs and spleens by using a MACS separation column (Miltenyi Biotec, Sunnyvale, CA, USA), before being pulsed with irradiated splenocytes loaded with OVA_257–264_ peptide. T cell activation was interpreted as the synthesis of IL-2 and IFN-γ by intracellular cytokine staining.

### 3.8. Cytokine Secretion, Cell Recovery and Proliferation

Cytokines were measured by ELISA, T cell survival in vitro was determined by the trypan blue exclusion assay and proliferation was measured in triplicate cultures by incorporation of ^3^H-thymidine (1 µCi/well; MP Biochemicals, Santa Ana, CA, USA) during the last 12 h of culture [16].

### 3.9. In Vitro Cytotoxicity Assay

Target cells (1 × 10^6^; B16-OVA) were stained with 5 μM CFSE (Biolegend, San Diego, CA, USA) in 1 mL of PBS followed by an incubation at 37 °C for 45 min. The cells were washed twice and re-suspended in 1 mL of medium followed by incubation at 37 °C for 30 min. T cells were added at different effector to target (E:T) cell ratios (1:5, 1:10 and 1:20). For estimation of the background, the wells containing target cells were run without the addition of T cells. The plates were incubated at 37 °C for 6 and 12 h before the assessment of cytotoxicity by flow cytometry. Prior to analysis, 15 μg/mL propidium iodide was added as a live/dead stain. The percentage of specific lysis was calculated as follows: cytotoxicity (%): [100% × dead target cells in experimental/(dead target cells in experimental sample + live target cells in experimental sample)] − [100% × dead target cells in target cell control/(dead target cells in target cell control + live target cells in target cell control)].

### 3.10. Xenograft Melanoma Model

Two weeks after adoptive stem cell transfer, mice were *s.c.* challenged on the flank with 4 × 10^6^ B16-OVA tumor cells in PBS or PBS without tumor cells as a control. The numbers of T cells were calculated based on total cell numbers in the spleen and draining LNs (inguinal, mesenteric and paraaortic) together with the percentages of CD8^+^TCRVβ5^+^ cells visualized using flow cytometry [6]. In some experiments, the mice were subsequently *i.p.* infected with 5 × 10^6^ PFU of VV-OVA and were *i.p.* given 20 ng rIL-2 (PeproTech, Rocky Hill, NJ, USA) after tumor inoculation twice per day for 3 days. The volume of the tumor (mm^3^) was measured using a caliper by a blinded investigator and calculated as follows: V = length × width^2^ × 0.52. Mice were sacrificed when the tumor size reached 20 mm in any direction.

### 3.11. Histology and Immunofluorescence

Mice were sacrificed on day 21 after melanoma challenge, before the tumor tissues were isolated and cut into several pieces. For general H&E staining, tumor tissues were fixed by 10% neutral formalin solution (VWR, West Chester, PA, USA), before the fixed samples were prepared and stained as described previously [6]. For immunofluorescent microscopy, tissues were frozen in cryovials on dry ice immediately after being resected. Later on, samples were prepared by cryosectioning and detailed protocol for immunofluorescent staining was described previously. In brief, FITC-labeled anti-TCRVβ5 antibody was used to detect the tumor-infiltrating OVA-specific HSC-CTLs.

### 3.12. Statistical Analysis

One-way ANOVA analysis was used for the statistical analysis between groups and significance was set at 5%. All statistics were calculated using GraphPad Prism (GraphPad Software, San Diego, CA, USA).

## 4. Conclusions

In the current study, we have demonstrated that a combination of genetic modification of HSCs and in vivo T cell development by Notch signaling facilitates the generation of tumor Ag-specific CTLs that suppressed melanoma growth in a murine model. Of note, mice receiving genetically modified HSCs and in vivo priming established persistent anti-tumor immunity. We believe that our strategy to generate naive tumor Ag-specific HSC-CTLs may be applied for the development of protective cancer vaccines.

## References

[1] Rosenberg S.A., Restifo N.P. (2015). Adoptive cell transfer as personalized immunotherapy for human cancer. Science.

[2] Koller K.M., Wang W., Schell T.D., Cozza E.M., Kokolus K.M., Neves R.I., Mackley H.B., Pameijer C., Leung A., Anderson B. (2016). Malignant melanoma-the cradle of anti-neoplastic immunotherapy. Crit. Rev. Oncol. Hematol..

[3] Soldner F., Hockemeyer D., Beard C., Gao Q., Bell G.W., Cook E.G., Hargus G., Blak A., Cooper O., Mitalipova M. (2009). Parkinson’s disease patient-derived induced pluripotent stem cells free of viral reprogramming factors. Cell.

[4] Raya A., Rodriguez-Piza I., Guenechea G., Vassena R., Navarro S., Barrero M.J., Consiglio A., Castella M., Rio P., Sleep E. (2009). Disease-corrected haematopoietic progenitors from fanconi anaemia induced pluripotent stem cells. Nature.

[5] Ebert A.D., Yu J., Rose F.F., Mattis V.B., Lorson C.L., Thomson J.A., Svendsen C.N. (2009). Induced pluripotent stem cells from a spinal muscular atrophy patient. Nature.

[6] Lei F., Zhao B., Haque R., Xiong X., Budgeon L., Christensen N.D., Wu Y., Song J. (2011). In vivo programming of tumor antigen-specific T lymphocytes from pluripotent stem cells to promote cancer immunosurveillance. Cancer Res..

[7] Haque R., Lei F., Xiong X., Bian Y., Zhao B., Wu Y., Song J. (2012). Programming of regulatory T cells from pluripotent stem cells and prevention of autoimmunity. J. Immunol..

[8] Haque M., Song J., Fino K., Sandhu P., Wang Y., Ni B., Fang D., Song J. (2016). Melanoma immunotherapy in mice using genetically engineered pluripotent stem cells. Cell Transplant..

[9] Haque M., Song J., Fino K., Sandhu P., Song X., Lei F., Zheng S., Ni B., Fang D., Song J. (2016). Stem cell-derived tissue-associated regulatory T cells ameliorate the development of autoimmunity. Sci. Rep..

[10] Li Z., Yang C.S., Nakashima K., Rana T.M. (2011). Small rna-mediated regulation of ips cell generation. EMBO J..

[11] Zhao T., Zhang Z.N., Rong Z., Xu Y. (2011). Immunogenicity of induced pluripotent stem cells. Nature.

[12] Haque M., Song J., Fino K., Wang Y., Sandhu P., Song X., Norbury C., Ni B., Fang D., Salek-Ardakani S. (2016). C-myc regulation by costimulatory signals modulates the generation of cd8+ memory T cells during viral infection. Open Biol..

[13] Sekine C., Koyanagi A., Koyama N., Hozumi K., Chiba S., Yagita H. (2012). Differential regulation of osteoclastogenesis by notch2/delta-like 1 and notch1/jagged1 axes. Arthritis Res. Ther..

[14] Kijima M., Yamaguchi T., Ishifune C., Maekawa Y., Koyanagi A., Yagita H., Chiba S., Kishihara K., Shimada M., Yasutomo K. (2008). Dendritic cell-mediated nk cell activation is controlled by jagged2-notch interaction. Proc. Natl. Acad. Sci. USA.

[15] Frankel T.L., Burns W.R., Peng P.D., Yu Z., Chinnasamy D., Wargo J.A., Zheng Z., Restifo N.P., Rosenberg S.A., Morgan R.A. (2010). Both cd4 and cd8 T cells mediate equally effective in vivo tumor treatment when engineered with a highly avid tcr targeting tyrosinase. J. Immunol..

[16] Zhao B., Song A., Haque R., Lei F., Weiler L., Xiong X., Wu Y., Croft M., Song J. (2009). Cooperation between molecular targets of costimulation in promoting T cell persistence and tumor regression. J. Immunol..

